# The impact of chemotherapy and survival prediction by machine learning in early Elderly Triple Negative Breast Cancer (eTNBC): a population based study from the SEER database

**DOI:** 10.1186/s12877-022-02936-5

**Published:** 2022-04-01

**Authors:** Kaiyan Huang, Jie Zhang, Yushuai Yu, Yuxiang Lin, Chuangui Song

**Affiliations:** 1 Department of Breast Surgery, Fujian Medical University Union Hospital, Fuzhou, 350001 Fujian Province China; 2 Department of General Surgery, Fujian Medical University Union Hospital, Gulou District, No.29, Xin Quan Road, Fuzhou, 350001 Fujian Province China; 3 Breast Cancer Institute, Fujian Medical University, Fuzhou, 350001 Fujian Province China

**Keywords:** Elderly triple negative breast cancer, Breast cancer-specific survival, Overall survival, SEER database, Machine learning

## Abstract

**Purpose:**

We aimed to analysis the impact of chemotherapy and establish prediction models of prognosis in early elderly triple negative breast cancer (eTNBC) by using machine learning.

**Methods:**

We enrolled 4,696 patients in SEER Database who were 70 years or older, diagnosed with primary early TNBC(larger than 5 mm), from 2010 to 2016. The propensity-score matched method was utilized to reduce covariable imbalance. Univariable and multivariable analyses were used to compare breast cancer-specific survival(BCSS) and overall survival(OS). Nine models were developed by machine learning to predict the 5-year OS and BCSS for patients received chemotherapy.

**Results:**

Compared to matched patients in no-chemotherapy group, multivariate analysis showed a better survival in chemotherapy group. Stratified analyses by stage demonstrated that patients with stage II and stage III other than stage I could benefit from chemotherapy. Further investigation in stage II found that chemotherapy was a better prognostic indicator for patients with T2N0M0 and stage IIb, but not in T1N1M0. Patients with grade III could achieve a better survival by receiving chemotherapy, but those with grade I and II couldn’t. With 0.75 in 5-year BCSS and 0.81 in 5-year OS for AUC, the LightGBM outperformed other algorithms.

**Conclusion:**

For early eTNBC patients with stage I, T1N1M0 and grade I-II, chemotherapy couldn’t improve survival. Therefore, de-escalation therapy might be appropriate for selected patients. The LightGBM is a trustful model to predict the survival and provide precious systemic treatment for patients received chemotherapy.

**Supplementary Information:**

The online version contains supplementary material available at 10.1186/s12877-022-02936-5.

## Introduction

Breast cancer is presently the commonest malignant tumor [1]. With the extension of average life expectancy of women, nearly 30%-40% of breast cancer patients were over 70 years old when they were initially diagnosed [2]. Moreover, by 2030, the number of elderly breast cancer might increase 57% in expectation [3]. However, due to the lack of clinical trial data for aged patients (enrollment rate not more than 20%), the management of senile breast cancer is still controversial, especially chemotherapy [4].

According to previous reports, the proportion of the elders who receive chemotherapy is much lower than that of the young patients, which may influence the prognosis in elders, particularly in triple negative breast cancer [5, 6]. A study based on SEER data-base showed higher breast cancer specific mortality in elderly triple negative breast cancer (eTNBC) when compared to younger cohort [7]. However, this difference is not significant when both of them received adjuvant treatment. Although the tumor biological characteristics in elders is higher hormone receptor(HR) expression, and less human epidermal growth factor receptor 2(Her2) expression, nearly 5% of them are eTNBC and account for 10%-20% TNBC in all ages [7–9]. Therefore, it is urgent to investigate the effect of chemotherapy in eTNBC and explore who could benefit from chemotherapy. There were two studies focused on this topic by using National Cancer Database (Jennifer A Crozier) and population from Swedish(Slavica Janeva) [9, 10]. Both of them recommended chemotherapy in general population. Nevertheless, there were still some insufficiencies in both studies. Neither of them compared the efficacy of chemotherapy in subgroups other than lymph node stage, for example, tumor size, different age groups, clinical stage and so on. Hence, we could not know who is more needed to receive chemotherapy. Even for the analysis of different nodal status, there is a contradiction. Jennifer draw a conclusion that chemotherapy should be recommended without considering nodal status. Yet, Slavica only found better results in nodal negative patients. Besides disease stage which is also an important predictor in elderly patients, the benefit of chemotherapy in elders depends on several added factors, such as comorbidities, tolerance to toxicities, heterogeneity in health, and expected life expectancy [11–14]. The discrete-time stochastic state transition simulation model showed that the benefit of chemotherapy is more common in the patients with higher risk, fewer comorbidities and longer expected survival [15]. Since life expectancy continues to improving in recent years, more patients might benefit from chemotherapy [16].

In order to make up for the insufficient evidence, we use the data from SEER data-base to conduct an analysis of the efficacy of chemotherapy in elderly TNBC by using propensity score matched(PSM). In addition, we investigated it in different subgroups according to stage, tumor size, lymph node status and histological grade. We also establish prediction models of the survival time of eTNBC by using machine learning. We believe our study may be helpful to predict the population benefit from chemotherapy in elderly breast cancer by combining the above methods.

## Materials and methods

### Ethics approval and consent to participate

Considering SEER database is publicly available and does not require informed patient consent. So, we did not need to get patient consent and exempt from Institutional Review Board approval. We signed a Data-Use Agreement for the SEER 1973–2016 Research Data File to get access conditions.

### Data source and study population

We used SEER*Stat version 8.3.8 to generate a case list. We enrolled 4,696 patients according to the following inclusion criteria: female; year of diagnosis from 2010 to 2016; age of diagnosis ≥ 70 years; breast carcinoma as the only primary malignant cancer diagnosis; American Joint Committee on Cancer (AJCC) sixth edition stage I-III; tumor larger than 5 mm in diameter; triple negative subtype. Patients who present with distant metastasis, in situ disease were expelled from the study. We defined two patient groups according to the *Chemotherapy recode* in SEER database: chemotherapy group or no-chemotherapy group. We calculated follow-up durations from January 1, 2010 to December 31, 2016. Patient characteristics and treatment courses in our study were identified, including age, race, marital status, surgery approach, chemotherapy status and radiotherapy status. Tumor characteristics included grade, AJCC stage, tumor status and nodal status.

### Outcome measurement

In our study, breast cancer-specific survival (BCSS) was used as a primary study outcome. It was calculated from the date of diagnosis to the date of death due to breast cancer. However, we should notice that if a patient died from other causes, the end date of her being followed-up in a BCSS analysis was the day of the last contact, the date of death from other causes, or the end of this study. Overall survival (OS), served as a secondary outcome, was defined as from the date of diagnosis to the date of death or was censored at the last follow-up date. Patients being lost to follow-up or survived at the end of the follow-up period were censored. If a patient still alive at the end of the follow-up period, the follow-up duration was calculated from the date of diagnosis to the end of this study. If a patient was lost to follow-up, the follow-up duration was calculated from the date of diagnosis to the day of the last contact.

### Statistical analysis

The chi-square test was conducted to describe the demographic and clinical characteristics of the chemotherapy and no-chemotherapy cases, in both the whole groups and 1:1 PSM groups. The Kaplan–Meier method was utilized to generate the survival curves. The log-rank test was conducted to identify whether the differences in BCSS or OS rates between chemotherapy patients and no-chemotherapy patients was statistically significant. Hazard ratio (HR) with 95% confidence intervals (CI) was calculated by using a Cox proportional hazard regression model to determine the outcome-related factors. Factors with a *P*-value of *0.05* or less in univariate analysis were included as candidate variables in the multivariate analysis. Proportional hazard assumptions were examined the by Schoenfeld residuals test. For the variables fail to meet the proportional hazards assumption, we conducted time-dependent covariate analysis to minimize the potential bias. In order to reduce the influences of baseline differences in demographic and clinical characteristics on outcome differences, 1:1 PSM method was performed to match patients in chemotherapy group and no-chemotherapy group. Covariables included in propensity score matching were age, race, marital status, grade, AJCC stage, tumor status, nodal status, surgery approach and radiation status. The two groups of patients were matched one to one by nearest-neighbor matching with a 0.1 caliper distance.

Before building machine learning models, all patients in the chemotherapy group were randomly divided to 2 sets, a training set and a testing set, at a 8:2 ratio. In the training set, K-nearest neighbor, CatBoost, decision tree, random forest method, Gradient Boost, LightGBM, neural network models, support vector machine and XGBoost models were developed to predict the 5-year BCSS and OS for patients in the chemotherapy group. The performance of these models was evaluated by ten‐fold cross validation.

These statistical analyses were conducted by using R software version 3.6.1 and Python Version 3.8. All statistical analyses were two-sided, and a *P* value of less than *0.05* was considered as a significance level.

## Results

### Demographics and clinical characteristics of the study population

Overall, 4,696 eligible patients were enrolled in our study, including 2,122 patients belonged to chemotherapy group and 2,574 patients belonged to no-chemotherapy group. The median follow-up time was 27 months. The baseline characteristics of the chemotherapy group and no-chemotherapy group were summarized in Table 1. There were significant differences in characteristics between two groups, including age, marital status, grade, AJCC stage, tumor status, nodal status and radiation status. The patients treated with chemotherapy presented a higher proportion of younger age (70–79 years old, 86.5% vs. 48.8%, *p* < 0.001), married status (married, 51.1% vs. 37.3%, *p* < 0.001), and grade III (81.7% vs. 72.5%, *p* < 0.001). A lower proportion of patients in chemotherapy group presented AJCC stage I, T1 stage and N0 stage (AJCC stage I, 33.3% vs. 51.2%, *p* < 0.001; T1 stage, 42.9% vs. 55.7%, *p* < 0.001; N0 stage, 60.8% vs. 79.2%, *p* < 0.001, respectively). In addition, the chemotherapy group were inclined to accept radiotherapy than no-chemotherapy group (55.6% vs. 39.4%, *p* < 0.001). Other characteristics, including race and surgery approach, were similarly distributed between two groups.

**Table 1:** Baseline characteristics of patients with chemotherapy and no-chemotherapy

**Characteristics**		**No-Chemotherapy (*n* = 2574)**		**Chemotherapy (*n* = 2122)**		**Total (*n* = 4696)**		***P***^c^
		**No**	**%**	**No**	**%**	**No**	**%**	
**Median follow-up (months) (IQR)**		27(12–49)		27(12–47)		27(12–48)		
**Age (years)**	**70–79**	1257	48.8	1836	86.5	3093	65.9	** < 0.001**
	**80 + **	1317	51.2	286	13.5	1603	34.1	
**Race**	**White**	2003	77.8	1636	77.1	3639	77.5	0.238
	**Black**	383	14.9	348	16.4	731	15.6	
	**Other**^a^	188	7.3	138	6.5	326	6.9	
**Marital status**	**Married**	959	37.3	1085	51.1	2044	43.5	** < 0.001**
	**Not married**^b^	1615	62.7	1037	48.9	2652	56.5	
**Grade**	**I and II**	707	27.5	388	18.3	1095	23.3	** < 0.001**
	**III**	1867	72.5	1734	81,7	3601	76.7	
**AJCC stage**	**I**	1317	51.2	706	33.3	2023	43.1	** < 0.001**
	**II**	960	37.3	999	47.1	1959	41.7	
	**III**	297	11.5	417	8.9	714	15.2	
**Tumor status**	**T1**	1435	55.7	910	42.9	2345	49.9	** < 0.001**
	**T2**	876	34.0	919	43.3	1795	38.2	
	**T3**	150	5.8	148	7.0	298	6.3	
	**T4**	113	4.4	145	6.8	258	5.5	
**Nodal status**	**N0**	2039	79.2	1290	60.8	3329	70.9	** < 0.001**
	**N1**	357	13.9	556	26.2	913	19.4	
	**N2**	104	4.0	172	8.1	276	5.9	
	**N3**	74	2.9	104	4.9	178	3.8	
**Surgery approach**	**No surgery**	150	5.8	107	5.0	257	5.5	0.239
	**Mastectomy and BCS**	2424	94.2	2015	95.0	4439	94.5	
**Radiation status**	**Yes**	1013	39.4	1179	55.6	2192	46.7	** < 0.001**
	**No**	1561	60.6	943	44.4	2504	53.3	

*Abbreviation: AJCC* American Joint Committee on Cancer, *BCS* Breast-conserving surgery, *IQR* Interquartile range

^a^Other includes American Indian/Alaskan native and Asian/Pacific Islander and Unknown

^b^Not married includes divorced, separated, single (never married), unmarried or domestic partner, and widowed

^c^The *P* value of the Chi-square test was calculated between the chemotherapy and no-chemotherapy groups, and bold type indicates significance

### Comparison of survival between chemotherapy group and no-chemotherapy group

The univariate Cox regression analysis for each variable was shown in Table S1. Compared to the survival of overall patients in no-chemotherapy group, the result of multivariate analysis shown in Table 2 revealed a better survival in patients received chemotherapy, according to BCSS and OS (HR = 0.656, 95% CI = 0.553–0.779, *p* < 0.001; HR = 0.561, 95% CI = 0.488–0.644, *p* < 0.001, respectively). We conducted 1:1 PSM analysis between patients in two groups to lower the effects of bias. Finally, we obtained a group with 2,660 patients, and each subgroup included 1,330 patients. As shown in Table 3, we performed the chi-square test for matched dataset. The P values for each covariables are more than 0.05, which indicates the propensity score overlapped well between the two groups of patients.

**Table 2:** Multivariate Cox proportional hazard model of breast cancer-specific survival (BCSS) and overall survival (OS) in all patients

Variables		BCSS		OS	
		**HR (95% CI)**	***P***	**HR (95% CI)**	***P***
**Age (years)**	**70–79**	Reference		Reference	
	**80 + **	1.315(1.111–1.557)	**0.001**	1.629(1.429–1.856)	** < 0.001**
**Marital status**	**Married**	Reference		Reference	
	**Not married**^a^	1.062(0.902–1.250)	0.472	1.120 (0.985–1.272)	0.084
**Race**	**White**	Reference		Reference	
	**Black**	1.068 (0.875–1.305)	0.516	1.070 (0.914–1.252)	0.399
	**Other**^b^	0.706 (0.507–0.983)	**0.039**	0.709 (0.549–0.916)	**0.008**
**Grade**	**I and II**	Reference		Reference	
	**III**	1.510 (1.222–1.865)	** < 0.001**	1.344(1.152–1.568)	** < 0.001**
**Stage**	**I**	Reference		Reference	
	**II**	3.982 (3.137–5.055)	** < 0.001**	2.602(2.221–3.048)	** < 0.001**
	**III**	11.609(9.015–14.949)	** < 0.001**	6.528(5.468–7.793)	** < 0.001**
**Surgery approach**	**No surgery**	Reference		Reference	
	**Mastectomy and BCS**	0.246(0.198–0.304)	** < 0.001**	0.293(0.245–0.351)	** < 0.001**
**Radiation**	**No**	Reference		Reference	
	**Yes**	0.626 (0.529–0.741)	** < 0.001**	0.565 (0.495–0.645)	** < 0.001**
**Chemotherapy**	**No**	Reference		Reference	
	**Yes**	0.656 (0.553–0.779)	** < 0.001**	0.561(0.488–0.644)	** < 0.001**

*Abbreviation: 70–79* 70–79 years old, *80* + More than 80 years old, *BCS* Breast Conserving Surgery, *HR* Hazard ratio

^a^Not married includes divorced, separated, single (never married), unmarried or domestic partner, and widowed

^b^Other includes American Indian/Alaskan native and Asian/Pacific Islander and Unknown. Bold type indicates significance

**Table 3:** Baseline characteristics of patients with chemotherapy and no-chemotherapy in PSM group

**Characteristics**		**No- Chemotherapy (*n* = 1330)**		**Chemotherapy (*n* = 1330)**		**Total (*n* = 2660)**		***P***^c^
		**No**	**%**	**No**	**%**	**No**	**%**	
**Median follow-up (months) (IQR)**		25(9–49)		26(11–48)		26(10–48.75)		
**Age (years)**	**70–79**	1046	78.6	1055	79.3	2101	79.0	0.703
	**80 + **	284	21.4	275	20.7	559	21.0	
**Race**	**White**	982	73.8	982	73.8	1964	73.8	1.000
	**Black**	248	18.7	248	18.7	496	18.7	
	**Other**^a^	100	7.5	100	7.5	200	7.5	
**Marital status**	**Married**	608	45.7	632	47.5	1240	46.6	0.371
	**Not married**^b^	722	54.3	698	52.5	1420	53.4	
**Grade**	**I and II**	259	19.5	287	21.6	546	20.5	0.195
	**III**	1071	80.5	1043	78.4	2114	79.5	
**AJCC stage**	**I**	647	48.6	665	50.0	1312	49.3	0.645
	**II**	494	37.1	491	36.9	985	37.0	
	**III**	189	7.1	174	13.1	363	13.6	
**Tumor status**	**T1**	727	54.7	723	54.4	1450	54.5	0.258
	**T2**	453	34.1	436	32.8	889	33.4	
	**T3**	83	6.2	80	6.0	163	6.1	
	**T4**	67	5.0	91	6.8	158	5.9	
**Nodal status**	**N0**	990	74.4	1030	77.4	2020	75.9	0.146
	**N1**	218	16.4	197	14.8	415	15.6	
	**N2**	67	5.0	66	4.9	133	5.0	
	**N3**	55	4.1	37	2.9	92	3.5	
**Surgery approach**	**No surgery**	70	5.3	67	5.0	137	5.2	0.861
	**Mastectomy and BCS**	1260	94.7	1263	95.0	2523	94.8	
**Radiation**	**Yes**	610	45.9	638	48.0	1248	46.9	0.294
	**No**	720	54.1	692	52.0	1412	53.1	

*Abbreviation: AJCC* American Joint Committee on Cancer; BCS, breast-conserving surgery, *IQR* Interquartile range

^a^Other includes American Indian/Alaskan native and Asian/Pacific Islander and Unknown

^b^Not married includes divorced, separated, single (never married), unmarried or domestic partner, and widowed

^c^The P value of the Chi-square test was calculated between the chemotherapy and no-chemotherapy groups, and bold type indicates significance

**Fig. 1:**
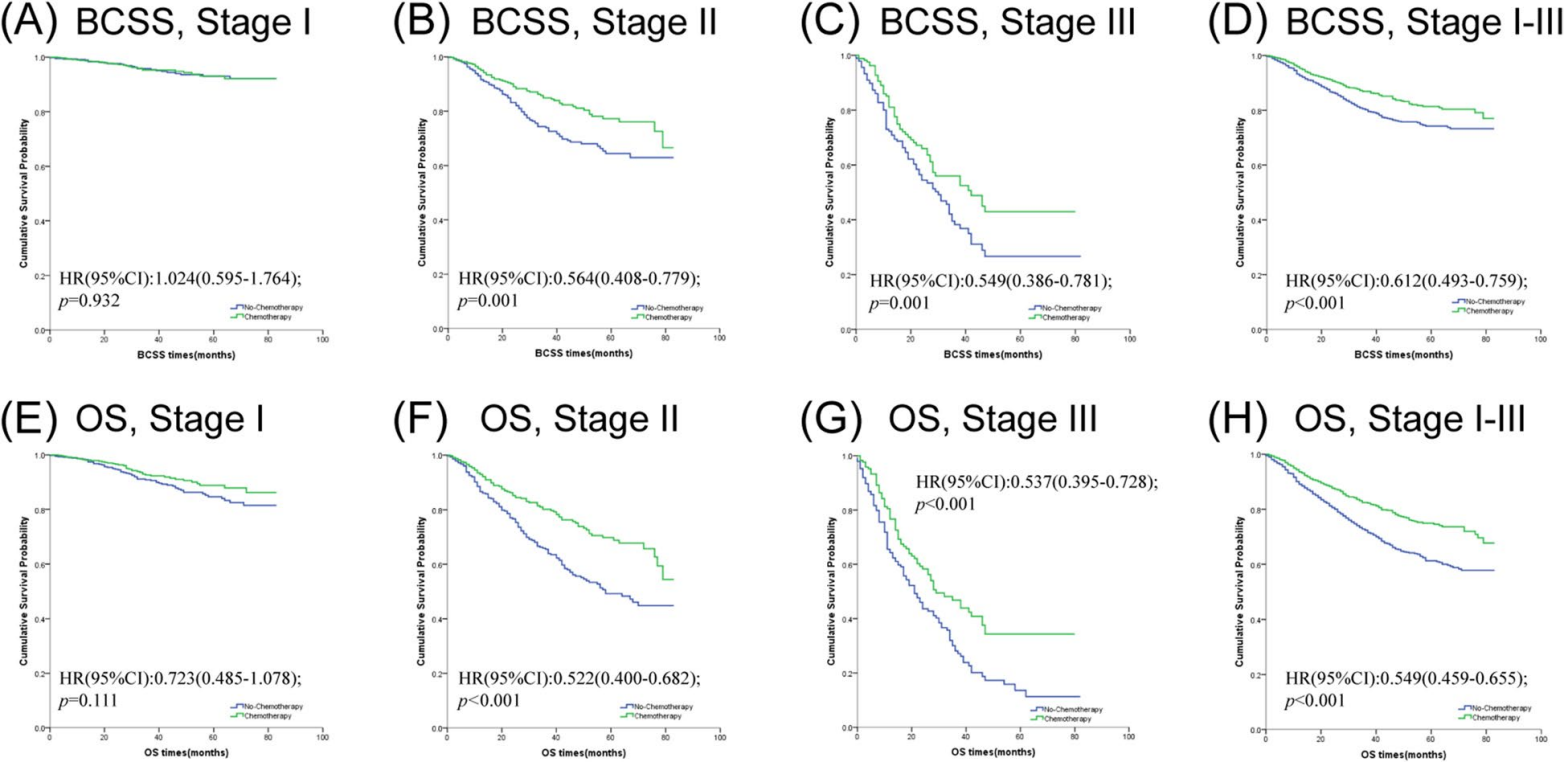
Kaplan–Meier survival curves of the effect of chemotherapy on BCSS (**A**–**D**) and OS (**E**–**H**) stratified by stage

**Table 4:** Comparison of breast cancer-specific survival (BCSS) and overall survival (OS) between matched patients with chemotherapy and no-chemotherapy in specific stage

Stage	BCSS			OS		
	**Events No**	**HRs (95%CI)**	***P*** ^a^	**Events No**	**HRs (95%CI)**	***P*** ^a^
**Stage I (** ***n*** ** = 1,312)**	53		0.932	103		0.111
** Chemotherapy**		1.024(0.595–1.764)			0.723(0.485–1.078)*	
** No-Chemotherapy**		Reference			Reference	
**Stage II (** ***n*** ** = 985)**	164		**0.001**	247		** < 0.001**
** Chemotherapy**		0.564(0.408–0.779)			0.522(0.400–0.682)*	
** No-Chemotherapy**		Reference			Reference	
**Stage III (** ***n*** ** = 363)**	142		**0.001**	192		** < 0.001**
** Chemotherapy**		0.549(0.386–0.781)			0.537(0.395–0.728)*	
** No-Chemotherapy**		Reference			Reference	
**Stage I-III (** ***n*** ** = 2,660)**	359		** < 0.001**	542		** < 0.001**
** Chemotherapy**		0.612(0.493–0.759)*			0.549(0.459–0.655)*	
** No-Chemotherapy**		Reference			Reference	

*Abbreviation*: *HR* Hazard ratio, *CI* Confidence interval, *BCSS* Breast cancer-specific survival, *OS* Overall survival, *Events No* Number of events

^a^*P* value was adjusted by a multivariate Cox proportional hazard regression model or a time-dependent covariate analysis. Bold type indicates significance

^b^The groups using time-dependent covariate analysis were specifically marked with asterisks(*)

In matched population, patients could significantly benefit from chemotherapy (BCSS, HR = 0.612, 95% CI = 0.493–0.759, *p* < 0.001; OS HR = 0.549, 95% CI = 0.459–0.655, *p* < 0.001, shown in Table 4). To investigate the effects of chemotherapy on patients with different subgroups, we stratified the patients by specific clinical features. We examined the proportional hazard assumptions for all subgroups. The results of the Schoenfeld residuals test for each subgroup were shown in Table S2 -Table S5. For the variables that fail to meet the proportional hazards assumption, we conducted time-dependent covariate analysis to minimize the potential bias. The subgroups, in which we conducted time-dependent covariate analysis, were specifically marked with asterisks.

**Fig. 2:**
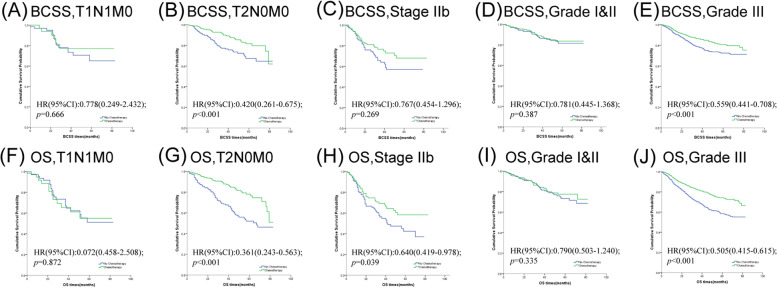
Kaplan–Meier survival curves of the effect of chemotherapy on BCSS (**A**-**E**) and OS (**F**-**J**) stratified by T stage, N stage and tumor grade

For the purpose of investigating the effects of chemotherapy on patients with different stages, we categorized the patients into stage I, stage II and stage III. The survival curves and results are shown in Fig. 1 and Table 4. As expected, chemotherapy didn’t lower the risk of cancer-specific mortality and all-cause mortality in stage I cohor (BCSS, HR = 1.024, 95% CI = 0.595–1.764, *p* = 0.932; OS HR = 0.723, 95% CI = 0.485–1.078, *p* = 0.111, respectively). But patients diagnosed with stage II can benefit from chemotherapy (BCSS, HR = 0.564, 95% CI = 0.408–0.779, *p* = 0.001; OS HR = 0.522, 95% CI = 0.400–0.682, *p* < 0.001, respectively). We observed similar phenomena in the stage III cohort(BCSS, HR = 0.549, 95% CI = 0.386–0.781, *p* < 0.001; OS HR = 0.537, 95% CI = 0.395–0.728, *p* < 0.001, respectively). But what made us curious was whether chemotherapy could be skipped in part of patients in stage II. We further investigated the effects of tumor status and nodal status in stage II cases between the two groups. As showcased in Fig. 2 and Table 5, we found that chemotherapy was a better prognostic indicator for patients with T2N0M0 (BCSS, HR = 0.420, 95% CI = 0.261–0.675, *p* < 0.001; OS HR = 0.361, 95% CI = 0.243–0.536, *p* < 0.001, respectively), but not for T1N1M0 (BCSS, HR = 0.778, 95% CI = 0.249–2.432, *p* = 0.666; OS HR = 1.072, 95% CI = 0.458–2.508, *p* = 0.872, respectively). The OS of stage IIb patients who were treated with chemotherapy was better than that without (HR = 0.640, 95% CI = 0419–0.978, *p* = 0.039). However, no significant difference in BCSS level was detected in patients who received chemotherapy compared with those who did not (HR = 0.767, 95% CI = 0.454–1.296, *p* = 0.321).

**Table 5:** Comparison of breast cancer-specific survival (BCSS) and overall survival (OS) between matched patients with chemotherapy and no-chemotherapy in specific clinical variables

Variables	BCSS			OS		
	**Events No**	**HRs (95%CI)**	***P***^a^	**Events No**	**HRs (95%CI)**	***P***^a^
**T1N1M0 (*n* = 109)**	21		0.666	34		0.872
** Chemotherapy**		0.778(0.249–2.432)			1.072(0.458–2.508)	
** No-Chemotherapy**		Reference			Reference	
**T2N0M0 (*n* = 595)**	80		** < 0.001**	123		** < 0.001**
** Chemotherapy**		0.420(0.261–0.675)			0.361(0.243–0.536)	
** No-Chemotherapy**		Reference			Reference	
**Stage IIb (*n* = 283)**	63		0.321	90		**0.039**
** Chemotherapy**		0.767(0.454–1.296)*			0.640(0.419–0.978)	
** No-Chemotherapy**		Reference			Reference	
**Grade I&II (*n* = 546)**	53		0.387	81		0.306
** Chemotherapy**		0.781(0.445–1.368)			0.790(0.503–1.240)*	
** No-Chemotherapy**		Reference			Reference	
**Grade III (*n* = 2,114)**	306		** < 0.001**	461		** < 0.001**
** Chemotherapy**		0.559(0.441–0.708)*			0.505(0.415–0.615)*	
** No-Chemotherapy**		Reference			Reference	

*Abbreviation*: *HR* Hazard ratio, *CI* Confidence interval, *BCSS* Breast cancer-specific survival, *OS* Overall survival, *Events No* Number of events

^a^*P* value was adjusted by a multivariate Cox proportional hazard regression model or a time-dependent covariate analysis. Bold type indicates significance

^b^The groups using time-dependent covariate analysis were specifically marked with asterisks(*)

Histological grade is one of the fundamental features to describe breast cancer. For patients with grade I and grade II, no statistical survival differences were identified between chemotherapy and no-chemotherapy patients (BCSS, HR = 0.781, 95% CI = 0.445–1.368, *p* = 0.387; OS HR = 0.790, 95% CI = 0.503–1.240, *p* = 0.306). While for patients with grade III, the chemotherapy patients demonstrated a better prognosis than no-chemotherapy patients in terms of both BCSS and OS (HR = 0.559, 95% CI = 0.441–0.708, *p* < 0.001; HR = 0.505, 95% CI = 0.415–0.615, *p* < 0.001, respectively).

### Machine-learning based outcome prediction in patients received chemotherapy

**Fig. 3:**
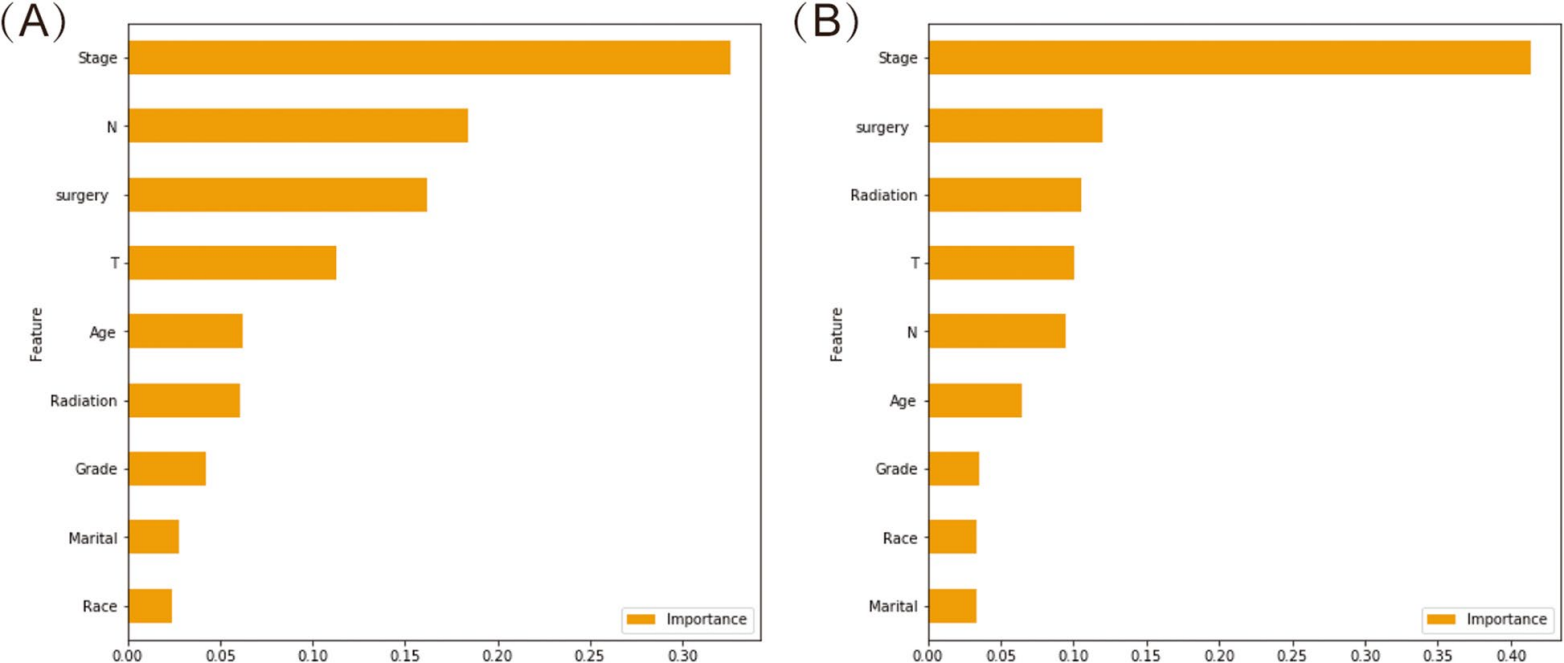
The importance score of predictor variables in predicting 5-year BCSS (**A**) and 5-year OS (**B**)

With respect to the nine algorithms for 5-year BCSS and 5-year OS, the performance metrics of the algorithms are presented in Table 6. The Table 7 showed the resulting confusion matrix. On average, the accuracy was 0.886 on 5-year BCSS and 0.857 on 5-year OS. The average precision of the examined ten algorithms was 0.888 on 5-year BCSS and 0.863 on 5-year OS. Similarly, the average sensitivity was 0.981 on 5-year BCSS and 0.969 on 5-year OS. There was average F1 score of 0.932 on 5-year BCSS and 0.913 on 5-year OS. In terms of the area under receiving operating characteristics curve (AUC), the highest AUC was observed in LightGBM. For predicting the 5-year BCSS, LightGBM achieved 0.882 accuracy, 0.887 precision, 0.991 sensitivity, 0.936 F1 score and 0.75 AUC. For 5-year OS, the parameters were 0.851, 0.859, 0.983,0.916 and 0.81 for accuracy, precision, sensitivity, F1 score and AUC, respectively. Considering all the parameters above, the LightGBM outperformed all other algorithms. The score of importance of each variable used in LightGBM was illustrated in Fig. 3, which demonstrated that the stage was the most relevant variables to explain the BCSS and OS. This model could provide more precious systemic treatments guidance and support for reducing overtreatment that may be present for patients with early eTNBC.

**Table 6:** Model performance for 5-year BCSS and 5-year OS

Algorithms	Accuracy	Precision	Sensitivity	F1 score	AUC
5-year BCSS					
K-nearest neighbor	0.879	0.882	0.98	0.928	0.70
Catboost	0.905	0.892	0.974	0.932	0.69
Decision tree	0.908	0.901	0.949	0.924	0.61
Random forest	0.869	0.889	0.971	0.929	0.70
Gradient booster	0.882	0.887	0.991	0.936	0.75
LightGBM	0.882	0.887	0.991	0.936	0.75
Neural network model	0.886	0.877	1.0	0.934	0.75
Support vector machine	0.882	0.887	0.991	0.936	0.51
XGBoost	0.879	0.892	0.98	0.934	0.70
5-year OS					
K-nearest neighbor	0.844	0.857	0.952	0.902	0.73
Catboost	0.877	0.86	0.977	0.915	0.76
Decision tree	0.882	0.869	0.940	0.903	0.69
Random forest	0.837	0.864	0.954	0.907	0.72
Gradient booster	0.849	0.855	0.985	0.916	0.80
LightGBM	0.851	0.859	0.983	0.916	0.81
Neural network model	0.86	0.877	0.949	0.911	0.79
Support vector machine	0.854	0.854	0.994	0.919	0.70
XGBoost	0.865	0.868	0.988	0.924	0.79

*Abbreviation*: *AUC* Area Under Curve

**Table 7:** Confusion matrix of nine algorithms for 5-year BCSS and 5-year OS

Algorithms		Predictions		Algorithms		Predictions	
		Dead	Alive			Dead	Alive
**5-year BCSS**				**5-year OS**			
**K-nearest neighbor**	Dead	3	47	**K-nearest neighbor**	Dead	15	56
	Alive	7	350		Alive	17	337
**Catboost**	Dead	8	42	**Catboost**	Dead	15	56
	Alive	9	348		Alive	8	346
**Decision tree**	Dead	13	37	**Decision tree**	Dead	21	50
	Alive	18	339		Alive	21	333
**Random forest**	Dead	7	43	**Random forest**	Dead	18	53
	Alive	10	347		Alive	16	338
**Gradient booster**	Dead	5	45	**Gradient booster**	Dead	12	59
	Alive	3	354		Alive	5	349
**LightGBM**	Dead	5	45	**LightGBM**	Dead	14	57
	Alive	3	354		Alive	6	348
**Neural network model**	Dead	0	50	**Neural network model**	Dead	24	47
	Alive	0	357		Alive	18	336
**Support vector machine**	Dead	5	45	**Support vector machine**	Dead	11	60
	Alive	3	354		Alive	2	352
**XGBoost**	Dead	8	42	**XGBoost**	Dead	18	53
	Alive	7	350		Alive	4	350

## Discussion

eTNBC is a special group of patients who are considered to have more indolent tumor behavior but higher risk of disease specific mortality when compared with younger patients [7, 17–20]. Insufficient treatment is considered as one of the reasons for this phenomenon. Surgery, chemotherapy and radiotherapy are the three primary treatments for eTNBC. Using PSM methods and multivariable regression in our study, we found all of them could reduce the disease-specific mortality and all-cause mortality in entire cohort. Compared with surgery and radiotherapy, chemotherapy leads to the most controversial. It was regarded as a double-edge sword in eTNBC due to its exclusive anti-tumor effect and high incidence of side-effects. Therefore, understanding how to optimally manage chemotherapy in eTNBC is increasingly important.

Several studies have focused on this topic. In accordance with us, most of them confirmed that chemotherapy could increase the survival rate in the general population of eTNBC [9, 10, 21, 22]. Meanwhile, they also pointed out the benefit was mainly observed in individuals who have low competing risks and who have high recurrent risks. However, presently there is no clear definition of high recurrent risk which can indicate the need of chemotherapy in eTNBC. Based on previous research conclusions, the status of lymph node was once regarded as a key point, among all clinical risk factors. Nearly all of those studies demonstrated the benefit of chemotherapy was only achieved in lymph nodes positive group [9, 10, 21, 22]. However, other clinicopathological factors have rarely been evaluated in those studies. Could different loads of lymph node metastasis, from only one involvement to more than nine, be put in the same planning, not to mention the difference of tumor burden, histological grades or other factors? In order to explore the value of chemotherapy in eTNBC with different clinicopathological characteristics, we investigated the efficacy of chemotherapy in more detailed subgroups.

Firstly, we found chemotherapy was necessary in patients with stage II and stage III, but not in stage I. Our conclusion is slightly different from that of Margaret M. Kozak who also worked based on SEER database [7]. They announced that people with stage II obtained greatest benefit from chemotherapy but not in stage III. And they believed this phenomenon was related to less intensive chemotherapy which was not effective enough in stage III TNBC. However, we could not agree with this conclusion. At first, SEER did not provide the detail of chemotherapy regimens, so we certainly could not know real intensity of chemotherapy for each patient. Secondly, Margaret M. Kozak compared the survival differences between the older group and younger group in different stages all receiving chemotherapy. They found chemotherapy could help to reduce the death risk in the elders to a level similar to that in the young in stage II but not in stage III. We thought they confused the effect of chemotherapy on the survival difference between the elders and young for the effect of chemotherapy on the survival of elders. After directly compared the risk between chemotherapy group and no-chemotherapy group, we found the effectiveness of reducing death risk by chemotherapy is consistent in stage II and stage III eTNBC. Therefore, we strongly recommended chemotherapy to stage III eTNBC. But what interests us is that does all patients in stage II need chemotherapy? What about the patients with negative lymph node or positive lymph node with small tumor? Further stratified analysis of the patients with stage II based on different N stage and T stage were conducted. We found that the patients with T1N1M0 could exempt chemotherapy, since no significant improvements were observed both in BCSS and OS after chemotherapy. But the patients with T2N0M0 and stage IIb could still benefit from chemotherapy. Besides, the relative risks of breast cancer specific mortality and overall mortality were reduced by about 25% ~ 35% in stage IIb. Therefore, we regarded that lymph node status should not be the only determinant. More detailed stratification could help us to identify candidates who really need chemotherapy which is particularly important in eTNBC.

Histological grade is an important pathological determinant for chemotherapy[23]. High grade always means high proliferation, poor prognosis and strong chemotherapy recommendation level [24]. In our study, we found that the risk of death decreased dramatically in grade III cohort after chemotherapy when compared with grade II and grade I cohorts. Considering chemotherapy is more effective in killing tumor with high proliferation, it could not be omitted in grade III eTNBC. Since there were no significant differences for their outcomes in grade I and grade II groups, we did not recommend chemotherapy to them. However, as showed in previous studies and our study, nearly 80% of eTNBC tumors presented as grade III, chemotherapy is still an important treatment modality.

At present, machine learning model can be considered as a model that automatically adjusts the weights of the factors. In addition, it can constructs a model that does not reduce the predictive effectiveness by fully exploiting data. On the contrary, some factors could not be incorporated into the model due to a lack of statistical significance in traditional statistics (for example, Cox proportional hazard regression model). In terms of performance, machine learning algorithms are more accurate than traditional statistical methods in predicting survival outcome in the fifth year. This is one of the purposes of our study. While Cox proportional hazards model is more appropriate for investigating the associations between covariates and end-point events. In terms of speed, machine learning algorithms can produce results within milliseconds. This strengths allows the system to react in real time. Delen and colleagues is the first to established a prediction model based on machine learning for patients of breast cancer [25]. Subsequently, machine learning is widely used in breast cancer. But, there is a paucity in machine learning algorithms predicting the impact of chemotherapy in early eTNBC. In our study, nine models were built to predict the 5-year BCSS and OS for patients received chemotherapy. Taken together, the results showed that the performance of LightGBM method exceeded that of all the other models in prediction of OS and BCSS. To the best of our knowledge, this is the first available predictive model for predicting survival impact of chemotherapy in early eTNBC, based on machine learning algorithms. We established the prediction model with the excellent performance. It could provide doctors with an easily accessible prediction tool and lead to more individualized and tailored chemotherapy for patients of early eTNBC.

In our study, we enrolled the largest number of participants to evaluate the value of chemotherapy in eTNBC by using the SEER database. After researching by PSM and investigating in more detailed subgroups, we believed we could offer a more helpful reference for clinical practice. However, we admit there are still several inevitable limitations. Since the SEER database did not provide information about comorbidity, we could not evaluate the impact of comorbidity on the results between the two groups. This might lead to minor bias. However, it is also a common inadequacy of those studies based on SEER databases. In general, breast cancer remains the most predominant cause of death in such groups of patients. By using an efficient modelling approach, we could effectively evaluate the prognostic impact of chemotherapy in elderly TNBC patients. Nevertheless, in view of the complexity of different comorbidity and the risk of it could not be quantified, we need to weight pros and cons carefully and individually. Another limitation is due to the SEER database itself, the information about the details of chemotherapy including drug, dose and number of cycles are unavailable. In this study, competing risk model might be an alternative statistical method, which can reduce the impact of competitive events on the results to a certain extent. But it might not be the best option. Because it may led to some inaccuracy and caused difficulties interpreting the results. Competing risk model is not necessarily better than the Cox proportional hazard model [26]. Thus, we didn't choose the competing risk model as the main statistical method. In contrast, Cox proportional hazard model is a more mature method. It could produce accurate results and it is more easier for Cox proportional hazard model to interpret the results than competing risk model. In addition, the models developed in this study have not been verified in the external validation cohort.

## Conclusion

In our study, chemotherapy improved survival in patients with grade III, T2N0M0, stage IIb and stage III early eTNBC. For patients diagnosed with stage I, T1N1M0, grade I and grade II, chemotherapy could not improve OS and BCSS. So, chemotherapy might be skipped. The nine models developed by machine learning performed well in survival prediction of early eTNBC patients and the LightGBM model have a best performance. The LightGBM is practical and trustful model to predict the survival and provide precious systemic treatment for patients in the chemotherapy group.

## Supplementary Information

**Additional file 1:** **Table S1.** Univariate Cox proportional hazard model of breast cancer-specific survival (BCSS) and overall survival (OS) in all patients.

**Additional file 2:** **Table S2.** The test of the proportional hazards assumption in subgroups sorted by specific stage (BCSS).

**Additional file 3:** **Table S3.** The test of the proportional hazards assumption in subgroups sorted by specific stage (OS).

**Additional file 4:** **Table S4.** The test of the proportional hazards assumption in subgroups sorted by specific clinical variables (BCSS).

**Additional file 5:** **Table S5.** The test of the proportional hazards assumption in subgroups sorted by specific clinical variables(OS).

## Data Availability

The datasets generated and analysed during the current study are available in the Surveillance, Epidemiology, and End Results (SEER) database. The URL of the database is https://seer.cancer.gov/.
