# Supplementary material for: The impact of chemotherapy and survival prediction by machine learning in early Elderly Triple Negative Breast Cancer (eTNBC): a population based study from the SEER database
Source: BMC Geriatr. 2022 Apr 1;22:268. doi: 10.1186/s12877-022-02936-5 (PMC8973884; doi:10.1186/s12877-022-02936-5)
Supplement: Supplementary file 1 — Additional file 1: Table S1. Univariate Cox proportional hazard model of breast cancer-specific survival (BCSS) and overall survival (OS) in all patients. [file 12877_2022_2936_MOESM1_ESM.docx]

**Table S1 :** Univariate Cox proportional hazard model of breast cancer-specific survival (BCSS) and overall survival (OS) in all patients.

| **Variables** | | **BCSS** | | |  | | **OS** | | | |  |
| --- | --- | --- | --- | --- | --- | --- | --- | --- | --- | --- | --- |
|  |  | **HR (95% CI)** | **P** | |  |  | **HR (95% CI)** | | **P** | |  |
| **Age (years)** | **70-79** | Reference |  | |  | | Reference | |  | |  |
|  | **80+** | 2.033(1.751-2.360) | **<0.001** | |  | | 2.583(2.300-2.901) | | **<0.001** | |  |
| **Marital status** | **Married** | Reference |  | |  | | Reference | |  | |  |
|  | **Not married ^a^** | 1.463 (1.253-1.709) | **<0.001** | |  | | 1.616 (1.431-1.826) | | **<0.001** | |  |
| **Race** | **White** | Reference |  | |  | | Reference | |  | |  |
|  | **Black** | 1.218 (1.000-1.482) | **0.050** | |  | | 1.170 (1.002-1.366) | | **0.047** | |  |
|  | **Other ^b^** | 0.856 (0.616-1.190) | 0.355 | |  | | 0.859 (0.667-1.108) | | 0.242 | |  |
| **Grade** | **I and II** | Reference |  | |  | | Reference | |  | |  |
|  | **III** | 1.871 (1.520–2.303) | **<0.001** | |  | | 1.532(1.318–1.781) | | **<0.001** | |  |
| **Stage** | **I** | Reference |  | |  | | Reference | |  | |  |
|  | **II** | 4.531 (3.593–5.715) | **<0.001** | |  | | 2.921(2.508–3.403) | | **<0.001** | |  |
|  | **III** | 13.417(10.567–17.035) | **<0.001** | |  | | 7.131(6.047–8.409) | | **<0.001** | |  |
| **Tumor status** | **T1** | Reference |  | |  | | Reference | |  | |  |
|  | **T2** | 3.660(3.020-4.436) | **<0.001** | |  | | 2.757(2.403-3.162) | | **<0.001** | |  |
|  | **T3** | 8.291(6.408-10.728) | **<0.001** | |  | | 5.189(4.222-6.378) | | **<0.001** | |  |
|  | **T4** | 10.960(8.509-14.118) | **<0.001** | |  | | 7.319(6.004-8.922) | | **<0.001** | |  |
| **Nodal status** | **N0** | Reference |  | |  | | Reference | |  | |  |
|  | **N1** | 3.111(2.609-3.709) | **<0.001** | |  | | 2.219(1.935-2.545) | | **<0.001** | |  |
|  | **N2** | 5.145(4.077-6.492) | **<0.001** | |  | | 3.428(2.829-4.156) | | **<0.001** | |  |
|  | **N3** | 8.337(6.563-10.590) | **<0.001** | |  | | 4.976(4.041-6.127) | | **<0.001** | |  |
| **Surgery** | **No surgery** | Reference |  | |  | | Reference | |  | |  |
| **approach** | **Mastectomy and BCS** | 0.134(0.109-0.164) | **<0.001** | |  | | 0.163(0.138-0.194) | | **<0.001** | |  |
| **Radiation** | **No** | Reference |  | |  | | Reference | |  | |  |
|  | **Yes** | 0.463(0.394-0.544) | **<0.001** | |  | | 0.415(0.365-0.471) | | **<0.001** | |  |
| **Chemotherapy** | **No** | Reference |  | |  | | Reference | |  | |  |
|  | **Yes** | 0.847 (0.728-0.986) | | **0.032** | |  | | 0.601(0.532-0.680) | | **<0.001** | |
|  |  |  |  | |  | |  | |  | |  |

Abbreviation: 70-79, 70-79 years old; 80+, more than 80 years old; BCS, Breast Conserving Surgery; HR, hazard ratio; ^a^ Not married includes divorced, separated, single (never married), unmarried or domestic partner, and widowed. ^b^ Other includes American Indian/Alaskan native and Asian/Paciﬁc Islander and Unknown. Bold type indicates significance.
