# Supplementary material for: The impact of chemotherapy and survival prediction by machine learning in early Elderly Triple Negative Breast Cancer (eTNBC): a population based study from the SEER database
Source: BMC Geriatr. 2022 Apr 1;22:268. doi: 10.1186/s12877-022-02936-5 (PMC8973884; doi:10.1186/s12877-022-02936-5)
Supplement: Supplementary file 2 — Additional file 2: Table S2. The test of the proportional hazards assumption in subgroups sorted by specific stage (BCSS). [file 12877_2022_2936_MOESM2_ESM.docx]

**Table S2:** The test of the proportional hazards assumption in subgroups sorted by specific stage (BCSS).

| Variables | P values from Schoenfeld residual test in subgroups | | | |
| --- | --- | --- | --- | --- |
|  | Stage I | Stage II | Stage III | Stage I-III |
| Age | 0.628 | 0.980 | 0.180 | 0.623 |
| Marital status | 0.527 | 0.720 | 0.870 | 0.759 |
| Grade | 0.995 | 0.670 | 0.200 | 0.338 |
| Race | 0.403 | 0.330 | 0.420 | 0.699 |
| AJCC stage | NA | NA | NA | 0.700 |
| Surgery approach | 0.054 | 0.230 | 0.140 | **0.027** |
| Chemotherapy status | 0.915 | 0.730 | 0.920 | 0.641 |
| Radiation status | 0.145 | 0.300 | 0.150 | **0.039** |
| Global | 0.411 | 0.800 | 0.250 | 0.280 |

Abbreviation: BCSS, breast cancer-specific survival; NA, not applicable.

Bold type indicates significance.
