# Supplementary material for: The impact of chemotherapy and survival prediction by machine learning in early Elderly Triple Negative Breast Cancer (eTNBC): a population based study from the SEER database
Source: BMC Geriatr. 2022 Apr 1;22:268. doi: 10.1186/s12877-022-02936-5 (PMC8973884; doi:10.1186/s12877-022-02936-5)
Supplement: Supplementary file 3 — Additional file 3: Table S3. The test of the proportional hazards assumption in subgroups sorted by specific stage (OS). [file 12877_2022_2936_MOESM3_ESM.docx]

**Table S3:** The test of the proportional hazards assumption in subgroups sorted by specific stage (OS).

| Variables | P values from Schoenfeld residual test in subgroups | | | |
| --- | --- | --- | --- | --- |
|  | Stage I | Stage II | Stage III | Stage I-III |
| Age | 0.431 | 0.299 | **0.028** | 0.207 |
| Marital status | 0.268 | 0.566 | 0.499 | 0.153 |
| Grade | 0.903 | 0.364 | 0.240 | 0.214 |
| Race | 0.827 | **0.031** | 0.596 | 0.316 |
| AJCC stage | NA | NA | NA | 0.166 |
| Surgery approach | 0.055 | 0.152 | **0.018** | **0.001** |
| Chemotherapy status | 0.994 | 0.910 | 0.733 | 0.913 |
| Radiation status | **0.006** | 0.075 | 0.071 | **<0.001** |
| Global | 0.101 | 0.121 | 0.025 | <0.001 |

Abbreviation: OS, overall survival; NA, not applicable.

Bold type indicates significance.
