# Supplementary material for: The impact of chemotherapy and survival prediction by machine learning in early Elderly Triple Negative Breast Cancer (eTNBC): a population based study from the SEER database
Source: BMC Geriatr. 2022 Apr 1;22:268. doi: 10.1186/s12877-022-02936-5 (PMC8973884; doi:10.1186/s12877-022-02936-5)
Supplement: Supplementary file 4 — Additional file 4: Table S4. The test of the proportional hazards assumption in subgroups sorted by specific clinical variables (BCSS). [file 12877_2022_2936_MOESM4_ESM.docx]

**Table S4:** The test of the proportional hazards assumption in subgroups sorted by specific clinical variables (BCSS).

| Variables | P values from Schoenfeld residual test in subgroups | | | | |
| --- | --- | --- | --- | --- | --- |
|  | T1N1M0 | T2N0M0 | Stage IIb | Grade I&II | Grade III |
| Age | 0.990 | 0.524 | 0.122 | 0.064 | 0.873 |
| Marital status | 0.390 | 0.640 | 0.618 | 0.088 | 0.667 |
| Grade | 0.630 | 0.922 | 0.619 | NA | NA |
| Race | 0.370 | 0.407 | 0.570 | 0.171 | 0.855 |
| AJCC stage | NA | NA | NA | 0.321 | 0.411 |
| Surgery approach | 0.420 | 0.148 | 0.802 | 0.934 | **0.015** |
| Chemotherapy status | 0.390 | 0.068 | 0.452 | 0.535 | 0.601 |
| Radiation status | 0.240 | 0.577 | **0.037** | 0.198 | 0.083 |
| Global | 0.700 | 0.251 | 0.235 | 0.236 | 0.278 |

Abbreviation: BCSS, breast cancer-specific survival; NA, not applicable;

Bold type indicates significance.
