# Supplementary material for: The impact of chemotherapy and survival prediction by machine learning in early Elderly Triple Negative Breast Cancer (eTNBC): a population based study from the SEER database
Source: BMC Geriatr. 2022 Apr 1;22:268. doi: 10.1186/s12877-022-02936-5 (PMC8973884; doi:10.1186/s12877-022-02936-5)
Supplement: Supplementary file 5 — Additional file 5: Table S5. The test of the proportional hazards assumption in subgroups sorted by specific clinical variables(OS). [file 12877_2022_2936_MOESM5_ESM.docx]

**Table S5:** The test of the proportional hazards assumption in subgroups sorted by specific clinical variables(OS).

| Variables | P values from Schoenfeld residual test in subgroups | | | | |
| --- | --- | --- | --- | --- | --- |
|  | T1N1M0 | T2N0M0 | Stage IIb | Grade I&II | Grade III |
| Age | 0.414 | 0.529 | 0.071 | **0.033** | 0.534 |
| Marital status | 0.912 | 0.225 | 0.425 | **0.020** | 0.634 |
| Grade | 0.836 | 0.331 | 0.239 | NA | NA |
| Race | 0.076 | 0.164 | 0.383 | 0.903 | 0.220 |
| AJCC stage | NA | NA | NA | 0.473 | 0.075 |
| Surgery approach | 0.467 | 0.320 | 0.772 | 0.622 | **0.001** |
| Chemotherapy status | 0.377 | 0.053 | 0.341 | 0.210 | 0.808 |
| Radiation status | 0.227 | 0.351 | 0.057 | 0.509 | **<0.001** |
| Global | 0.294 | 0.148 | 0.152 | 0.059 | 0.001 |

Abbreviation: OS, overall survival; NA, not applicable.

Bold type indicates significance.
